# Factors associated with intention to undergo elective oocyte cryopreservation among unmarried nurses in South Korea: a cross-sectional study

**DOI:** 10.4069/whn.2026.08.18

**Published:** 2026-09-29

**Authors:** Danbi Oh, Moonjeong Kim

**Affiliations:** 1 Rehabilitation Medicine Ward, Pusan National University Hospital, Busan, Korea; 2 Department of Nursing, Pukyong National University, Busan, Korea

**Keywords:** Attitude, Cryopreservation, Decision making, Nurses, Oocytes

## Abstract

**Purpose:**

This study examined intention to undergo elective oocyte cryopreservation (EOC) among unmarried nurses younger than 40 years and identified factors associated with EOC intention.

**Methods:**

This descriptive correlational study used a cross-sectional survey design and was conducted from September 12 to September 30, 2025 at a tertiary hospital in Busan, South Korea. The participants were 159 unmarried nurses. Attitudes toward EOC, procedural burden, ethical concerns, health-related concerns, and EOC knowledge were measured using a modified Fertility Preservation Survey. EOC intentions were assessed using a visual analog scale. Descriptive statistics, independent t-tests, Pearson correlation analysis, and multiple linear regression analyses were performed.

**Results:**

Participants reported a moderate level of EOC intention. EOC intention differed significantly by age, with nurses in their 20s reporting higher scores than those in their 30s (t=3.19, *p*=.002). EOC intention was positively correlated with attitudes toward EOC (r=.53, *p*<.001) and negatively correlated with health-related concerns (r=–.26, *p*=.001). In multiple linear regression analysis, greater EOC intention was significantly associated with more positive attitudes toward EOC (β=.47, *p*<.001), lower health-related concerns (β=–.23, *p*=.003), and younger age (β=–.21, *p*=.009). The model explained 32.0% of the variance.

**Conclusion:**

Early, evidence-based education and counseling that strengthen positive attitudes toward EOC and directly address health-related concerns may help unmarried nurses make informed reproductive decisions.

## Introduction

In South Korea, marriage is increasingly viewed as a personal choice, and delayed marriage has become more common [1]. In 2024, the mean age at first marriage among South Korean women was 31.6 years [2], higher than the Organization for Economic Co-operation and Development average of 30.7 years [3]. Because female reproductive capacity declines with age, delaying attempts to conceive increases the risk of infertility [4]. Consistent with this pattern, the reported rate of infertility diagnosis among South Korean women increases with age, from 14.2% among women younger than 29 years to 17.4% among those aged 30–34 years, 28.8% among those aged 35–39 years, and 37.9% among those aged 40 years or older [5].

Ovarian reserve, commonly assessed using anti-Müllerian hormone levels, is a key indicator of female reproductive capacity [6]. Ovarian reserve generally declines after the mid-30s [6], and advancing age is recognized as a major, unmodifiable risk factor for infertility [4]. This age-related decline in fertility may limit women’s ability to plan pregnancy and childbirth at their preferred time and may contribute to the psychological and financial burdens associated with infertility diagnosis and the use of assisted reproductive technology (ART) [7,8]. In this context, fertility preservation is understood as a strategy for broadening women’s reproductive choices and life-planning autonomy in light of age-related biological constraints [9].

The main fertility preservation methods include oocyte cryopreservation and embryo cryopreservation [10]. Oocyte cryopreservation has been used increasingly because individuals can choose it independently [9]. It is classified as medically indicated oocyte cryopreservation or nonmedical elective oocyte cryopreservation (EOC) [11,12]. Historically, oocyte cryopreservation was limited to circumstances in which ovarian function was expected to decline because of treatments such as chemotherapy [13]. More recently, however, interest in and use of EOC have increased alongside delays in marriage and childbirth [14]. In the United States, the number of EOC procedures reportedly increased from 4,153 in 2014 to 16,436 in 2021 [14]. In South Korea, however, EOC decision-making is constrained by sociocultural norms that childbirth should occur within marriage [1], low social acceptance, limited institutional support [15], and high costs [16]. Identifying EOC intention and the factors associated with it is therefore important for understanding and supporting women’s reproductive decision-making.

Previous studies have reported that EOC intention is associated with sociodemographic characteristics, including age, income, and educational level [17-19]; cognitive factors, including EOC knowledge and attitudes [20]; and perceived barriers, including financial burden, procedural burden, ethical concerns, and health-related concerns [7,21]. Positive attitudes toward EOC, in particular, have repeatedly been identified as closely associated with EOC intention [20]. Taken together, these findings suggest that EOC intention reflects a decision-making process shaped by both cognitive appraisal and perceived risk. However, previous studies have primarily examined motives, perceptions, experiences, or knowledge regarding EOC [17-19,21], and few have comprehensively analyzed the relative influence of multiple factors associated with EOC intention.

Nurses are routinely exposed to shift work, high job stress, irregular schedules, prolonged standing, and repetitive physical labor. These occupational factors have been associated with reproductive health problems, including disrupted ovulatory cycles, hormonal imbalance, and irregular menstruation [22-24]. Studies of female healthcare professionals have also reported higher rates of infertility and ART use than those in the general female population [25]. In particular, female surgeons often postpone pregnancy and childbirth during training and career development, and higher rates of ART use have been reported in this group [25]. Women with strong career-development aspirations have also been reported to be more likely to choose EOC to preserve future fertility [26]. Nurses likewise work in a predominantly female healthcare profession, and many pursue further education and professional advancement to support career development and occupational stability [27]. In addition, shift work, job stress, and other occupational demands may shape decisions about childbirth timing and fertility preservation. Identifying EOC intention and its associated factors is therefore essential to support informed decision-making about reproductive health and future family planning among unmarried nurses.

To date, EOC-related studies have focused mainly on the general female population or college students [7,17,19], whereas research on unmarried nurses remains scarce. This study therefore aimed to identify the level of EOC intention and the factors associated with it among unmarried nurses. The findings are intended to provide foundational evidence for education and counseling strategies that help nurses make informed reproductive decisions.

The specific objectives of this study are as follows:

• To analyze participants’ general characteristics and differences in EOC intention according to those characteristics.

• To determine participants’ attitudes toward EOC, procedural burden, ethical concerns, health-related concerns, and EOC knowledge.

• To identify correlations among EOC intention, attitudes toward EOC, procedural burden, ethical concerns, health-related concerns, and EOC knowledge.

• To identify factors associated with participants’ EOC intention.

## Methods

**Ethics statement:**This study was approved by the Institutional Review Board of Pukyong National University (No. PKNU IRB 2025-07-002). Informed consent was obtained from the participants.

### Study design

This study used a descriptive correlational design to identify EOC intention and associated factors among unmarried female nurses younger than 40 years. The study was reported in accordance with the STROBE (Strengthening the Reporting of Observational Studies in Epidemiology) reporting guidelines [28].

### Participants

Participants were unmarried female nurses younger than 40 years who were employed at Pusan National University Hospital in Busan, Korea. The age limit was set at younger than 40 years on the basis of age-related fertility decline and the clinical characteristics of EOC. Female fertility has been reported to decline gradually after 32 years of age and sharply after 37 years of age, with pregnancy success rates decreasing substantially after 40 years of age [29]. In addition, age at the time of EOC is an important determinant of future pregnancy success, and previous studies have recommended performing the procedure before 40 years of age [30].

Participants were recruited by convenience sampling if they understood the study purpose and voluntarily agreed to participate. The inclusion criteria were as follows: (1) being an unmarried female nurse working at a tertiary hospital, (2) being 23 to 39 years of age, and (3) voluntarily consenting to participate. The exclusion criterion was prior experience with oocyte cryopreservation. During recruitment, the study objectives and eligibility criteria were explained in advance, and the same criteria were applied to all participants.

The sample size was calculated using G*Power 3.1.9.7 (Heinrich Heine University Düsseldorf, Düsseldorf, Germany). Eleven independent variables were specified: six general characteristics (age, educational level, position, monthly income, religion, and experience with obstetric and gynecologic consultations) and five main variables (attitudes toward EOC, procedural burden, ethical concerns, health-related concerns, and EOC knowledge). The significance level was set at α=.05, and statistical power was set at 1−β=.90. Although previous studies of EOC intention reported significant differences according to sociodemographic characteristics such as age [19], they did not provide statistics that could be directly used or converted to estimate an effect size for sample size calculation. Therefore, Cohen’s medium effect size of f2=.15 was applied [31], yielding a minimum required sample size of 152 participants. After a 5% dropout rate was considered, questionnaires were distributed to 159 individuals. All returned questionnaires were included in the analysis, resulting in a final sample of 159 participants, which met the minimum required sample size.

### Measurements

The independent variables—attitudes toward EOC, procedural burden, ethical concerns, health-related concerns, and EOC knowledge—were measured using a modified and supplemented version of the Fertility Preservation Survey (FPS) developed by Daniluk and Koert [7]. The FPS was developed to assess unmarried women’s perceptions of and factors related to fertility preservation. It consists of 44 items covering attitudes toward fertility preservation, factors influencing oocyte cryopreservation, and fertility-preservation knowledge.

Permission to use the instrument was obtained from the original authors. The instrument was translated into Korean by a specialist in English language and literature. Two professors specializing in women’s health nursing then reviewed and revised the translation for the appropriateness of reproductive-health and oocyte-cryopreservation terminology and the content validity of the items. A bilingual specialist in English language and literature subsequently performed a back-translation, and semantic equivalence was verified by comparing the original instrument with the back-translated version. To reflect the Korean healthcare context, the item on oocyte cryopreservation costs in Canada was revised to approximately 2.5 million to 5.0 million Korean won (KRW), using the domestic procedure-cost range provided by the Seoul Metropolitan Government as a reference [32]. The revised items were finalized after an additional review by the two professors specializing in women’s health nursing.

In this study, the attitude and knowledge items from the original FPS were used, and exploratory factor analysis was conducted to derive independent variables from the items on factors influencing oocyte cryopreservation. The suitability of the data for factor analysis was supported by a Kaiser-Meyer-Olkin value of .80 and a significant Bartlett test of sphericity (*p*<.001). Principal component analysis with varimax rotation extracted three factors with eigenvalues of 1 or greater; these factors explained 60.7% of the total variance. The factors were named according to their conceptual meaning and the item with the highest factor loading. The first factor, centered on the item “the possibility of having to repeat the procedure to increase the chances of pregnancy,” was named “procedural burden.” The second factor, centered on the item “concerns about the ethical and moral issues of oocyte cryopreservation,” was named “ethical concerns.” The third factor, centered on the item “concerns that the child may have health problems if born using frozen oocytes,” was named “health-related concerns.” These subfactors were treated as separate independent constructs and entered as individual independent variables; no total score was calculated. This approach was used because each subfactor reflected a distinct concept and because the analysis aimed to estimate the separate association of each subfactor with EOC intention.

#### Elective oocyte cryopreservation attitude

Attitudes toward EOC were measured using the attitude domain of the FPS. This domain consists of 11 items assessing the extent to which EOC is perceived as a fertility-preservation strategy, awareness of the need for financial, institutional, and informational support, and attitudes toward posthumous donation of frozen oocytes. Example items include “If a woman is not ready to have children in her 20s or 30s, she should consider preserving her fertility through oocyte cryopreservation,” “If oocyte cryopreservation is needed, the cost should be supported by health insurance or the public healthcare system,” and “If social discussions and an institutional foundation for oocyte donation are established, I intend to participate in a donation program utilizing oocyte cryopreservation.” Each item was rated on a 4-point Likert scale ranging from 1 (“strongly disagree”) to 4 (“strongly agree”). Item scores were summed to calculate a total score ranging from 11 to 44, with higher scores indicating more positive and accepting attitudes toward oocyte cryopreservation. Cronbach’s α was .70 in the study by Akhondi et al. [33] and .80 in this study.

#### Procedural burden

Procedural burden refers to the financial burden, possibility of repeated procedures, procedural discomfort, and concerns about success rates that may accompany elective oocyte cryopreservation. This subscale consists of four items. Example items include “the possibility of having to repeat the procedure to increase the chances of pregnancy,” “the discomfort or side effects that may occur during hormone administration and oocyte retrieval,” and “the total cost required for the procedure.” Each item was rated on a 4-point Likert scale ranging from 1 (“does not influence at all”) to 4 (“strongly influences”). Item scores were summed to calculate a total score ranging from 4 to 16, with higher scores indicating greater procedural burden. In this study, Cronbach’s α for this subscale was .71.

#### Ethical concerns

Ethical concerns refer to apprehension about ethical and moral issues and social perceptions associated with elective oocyte cryopreservation. This subscale consists of four items. Example items include “concerns about the ethical and moral issues of oocyte cryopreservation,” “worries about the negative views or judgments of those around me,” “concerns about intervening in the natural pregnancy process,” and “the burden of deciding how to dispose of unused oocytes.” Each item was rated on a 4-point Likert scale ranging from 1 (“does not influence at all”) to 4 (“strongly influences”). Item scores were summed to calculate a total score ranging from 4 to 16, with higher scores indicating greater ethical concerns. In this study, Cronbach’s α for this subscale was .73.

#### Health-related concerns

Health-related concerns refer to worries about the potential effects of elective oocyte cryopreservation on the health of the woman and offspring. This subscale consists of two items: “concerns that the child may have health problems if born using frozen oocytes” and “the long-term risks that oocyte retrieval may pose to future health or fertility.” Each item was rated on a 4-point Likert scale ranging from 1 (“does not influence at all”) to 4 (“strongly influences”). Item scores were summed to calculate a total score ranging from 2 to 8, with higher scores indicating greater health-related concern. Cronbach’s α for this subscale was .68. Because the health-related concerns subscale consisted of only two items, Cronbach’s α alone was limited as an index of internal consistency. Therefore, inter-item correlation was additionally analyzed, revealing a significant, moderate positive correlation between the two items (r=.52, *p*<.001), which indicated an acceptable level of item relatedness.

#### Elective oocyte cryopreservation knowledge

EOC knowledge was measured using the knowledge domain of the FPS. This section evaluates the degree of understanding regarding the relationship between a woman’s age and fertility, the procedures and effects of oocyte cryopreservation, and information related to fertility preservation, encompassing a total of 12 items. Examples include “For women over 30 years of age, fertility can be influenced by health status and lifestyle in addition to age,” and “A sufficient number of oocytes can always be secured with a single procedure for oocyte cryopreservation.” Each item was scored 1 point for a correct answer and 0 points for an incorrect answer, with total scores ranging from 0 to 12. A higher score denotes a higher level of EOC knowledge. Because the knowledge items are structured as true/false questions, internal consistency reliability was not reported in previous studies. In this study, the calculated Kuder-Richardson 20 coefficient was .66.

#### Elective oocyte cryopreservation intention

EOC intention was measured using a visual analog scale (VAS). Participants were asked to rate “the degree of willingness to choose oocyte cryopreservation before reproductive capacity further declines, in the event of delayed marriage” on a scale from 0 to 10. Although previous studies have often measured EOC intention as a dichotomous variable [20], no standardized tool for measuring EOC intention as a continuous variable has been reported. Therefore, this study used a VAS to measure the degree of intention in greater detail. Respondents indicated their intention on a scale from 0 (“no intention at all”) to 10 (“will definitely do it”), with higher scores indicating stronger EOC intention.

#### General characteristics

To identify background factors related to EOC, participants were asked about sociodemographic characteristics previously reported to be associated with EOC intention, including age, educational level, position, monthly income, and religion [17-19]. Age was categorized as 23–29 years or 30–39 years. This categorization was based on previous findings that women perceive a need to receive information about elective oocyte cryopreservation before 30 years of age [16] and on research suggesting that approximately 30 years of age is a clinically important time point for counseling and information provision because procedural efficiency declines with age [34]. The questionnaire also included items on prior obstetric and gynecologic consultation, including whether participants had received such care and the examination results [27]. Participants who had received obstetric and gynecologic consultations could provide multiple responses regarding the reasons for consultation and the examination results.

### Data collection

Data were collected from September 12 to September 30, 2025, at Pusan National University Hospital in Busan, Korea. Before data collection, the study purpose and procedures were explained to the nursing department, and departmental cooperation was obtained. A recruitment notice was then posted on the hospital intranet bulletin board, which was accessible only to hospital staff. The notice described the study objectives, inclusion criteria, and exclusion criteria.

The survey was administered online using Google Forms (Google LLC, Mountain View, CA, USA), and participants accessed it through a URL or QR code. Before beginning the survey, participants viewed the informed consent form and screening questions. Only those who voluntarily agreed to participate and met the inclusion criteria could proceed to the questionnaire. The questionnaire also included items related to participant eligibility, including sex, age, marital status, occupational category, and previous experience with oocyte cryopreservation, to reconfirm that respondents met the study criteria.

The survey was configured so that participants had to answer each item before moving to the next, thereby preventing incomplete responses. As a result, there were no missing values. The researcher’s phone number, email address, and departmental affiliation were provided so that participants could ask questions during participation. Completing the questionnaire took approximately 10–15 minutes, and all participants received a mobile gift certificate worth 5,000 KRW as a token of appreciation.

### Data analysis

The collected data were analyzed using IBM SPSS Statistics ver. 28.0 (IBM Corp., Armonk, NY, USA). Participants’ sociodemographic characteristics and main variables were summarized using descriptive statistics, including frequencies, percentages, means, and standard deviations. The normality of the main variables was assessed using skewness and kurtosis. The criteria proposed by Kline [35]—an absolute skewness value less than 3 and an absolute kurtosis value less than 10—were used to evaluate normality. The normality of the regression model was examined using a histogram of standardized residuals and a normal P-P plot. Differences in EOC intention according to general characteristics were analyzed using independent t-tests, and correlations among the main variables were examined using Pearson correlation coefficients.

To identify factors associated with EOC intention, multiple linear regression analysis was performed using the enter method. Regression assumptions were evaluated before interpretation of the model. Multicollinearity was assessed using tolerance values, with tolerance >0.1, and variance inflation factors (VIFs), with VIF <10. Independence of residuals was evaluated using the Durbin-Watson statistic.

## Results

### Differences in intention toward elective oocyte cryopreservation according to the general characteristics of unmarried nurses

Participants’ general characteristics and differences in EOC intention according to these characteristics are shown in Table 1. The age distribution was nearly even: 78 participants (49.1%) were aged 23–29 years, and 81 (50.9%) were aged 30–39 years. Most participants had a bachelor’s degree (n=129, 81.1%) and were staff nurses (n=156, 98.1%). Monthly income was less than 3 million KRW for 89 participants (56.0%) and 3 million KRW or more for 70 participants (44.0%). A total of 110 participants (69.2%) reported no religious affiliation, and 137 (86.2%) had previously received obstetric and gynecologic care. Among participants with prior obstetric and gynecologic visits, the reported reasons for consultation were gynecologic problems (136 responses, 99.3%), routine check-ups (88 responses, 64.2%), and obstetric problems (seven responses, 5.1%). Reported examination findings were most commonly normal findings (82 responses, 59.9%), followed by polycystic ovary syndrome (31 responses, 22.6%), uterine fibroids (19 responses, 13.9%), and endometriosis (nine responses, 6.6%). Other responses included ovarian cysts, genital tract inflammation, and amenorrhea. 

The analysis of EOC intention according to general characteristics showed that participants aged 23–29 years had significantly higher EOC intention scores than those aged 30–39 years (6.11±2.50 vs. 4.73±2.91; t=3.19, *p*=.002). By contrast, EOC intention did not differ significantly according to educational level, position, monthly income, religion, or prior obstetric and gynecologic care.

### Descriptive statistics of main variables related to elective oocyte cryopreservation among unmarried nurses

Descriptive statistics for the main variables are presented in Table 2. The mean scores were 27.31±6.06 for attitudes toward EOC, 13.47±2.06 for procedural burden, 9.49±2.92 for ethical concerns, 5.73±1.57 for health-related concerns, 8.45±1.45 for EOC knowledge, and 5.38±2.78 for EOC intention. The absolute skewness values for all variables were less than 3, and the absolute kurtosis values were less than 10, indicating that the variables met the normality criteria.

### Correlations among elective oocyte cryopreservation-related variables and intention toward EOC among unmarried nurses

EOC intention was positively correlated with attitudes toward EOC (r=.53, *p*<.001). In analyses of the remaining EOC-related variables, EOC intention was negatively correlated with health-related concerns (r=–.26, *p*=.001) but was not significantly correlated with procedural burden, ethical concerns, or EOC knowledge. Correlation coefficients among the independent variables were all less than .80, suggesting a low likelihood of multicollinearity (Table 3).

### Factors associated with intention toward elective oocyte cryopreservation among unmarried nurses

Before the multiple regression analysis of EOC intention was interpreted, the assumptions of regression were examined. The Durbin-Watson statistic was 1.82, supporting the independence of residuals. Tolerance values ranged from 0.68 to 0.97, and VIFs ranged from 1.03 to 1.47, indicating no evidence of problematic multicollinearity. In addition, the histogram of standardized residuals approximated a normal distribution, and the observed values in the normal P-P plot were generally close to the diagonal line, supporting the normality of residuals.

Regression coefficients showed that attitudes toward EOC (β=.47, *p*<.001), health-related concerns (β=–.23, *p*=.003), and age (β=–.21, *p*=.009) were significantly associated with EOC intention. Specifically, more positive attitudes toward EOC and lower health-related concerns were associated with greater EOC intention. For age, participants aged 30–39 years had lower EOC intention than those aged 23–29 years, which served as the reference group. On the basis of the absolute standardized regression coefficients, attitudes toward EOC had the strongest association with EOC intention, followed by health-related concerns and age. The regression model was statistically significant (F=7.70, *p*<.001), and the adjusted coefficient of determination was .32, indicating that the model explained 32.0% of the variance in EOC intention (Table 4).

## Discussion

This study examined EOC intention and associated factors among unmarried nurses. Attitudes toward EOC, health-related concerns, and age were significantly associated with EOC intention. These findings suggest that EOC intention reflects a decision-making process shaped by cognitive appraisal, perceived health risks, and life-course factors. Accordingly, efforts to understand EOC intention among unmarried nurses should consider these factors together rather than in isolation.

First, attitudes toward EOC had the highest standardized regression coefficient, indicating the strongest association with EOC intention. This finding is consistent with previous studies reporting that positive attitudes toward EOC are associated with EOC intention [18] and supports the theory of planned behavior (TPB), in which attitude is a core determinant of behavioral intention [36]. According to the TPB, behavioral intention is determined by attitude, subjective norms, and perceived behavioral control; among these constructs, attitude has consistently been reported as an important predictor of health-related behavioral intentions [36,37]. Because EOC is not merely a decision about using a medical service but also a value-based decision about future life planning and family formation, women who evaluate EOC positively may be more likely to view it as a realistic fertility-preservation option. Previous studies have reported that some women perceive EOC as a medical intervention for specific circumstances rather than as a preventive strategy [18], and these differences in perception may shape both attitudes toward EOC and intention to undergo the procedure. Women who had considered or undergone EOC also tended to understand EOC-related information better than the general female population [33], and the amount and quality of information provided by healthcare professionals and mass media have been reported to affect women’s perceptions and attitudes [33]. Therefore, structured education and information programs are needed to help women understand EOC as a fertility-preservation option and make decisions based on adequate evidence-based information.

In this study, unmarried nurses had lower attitudes toward EOC scores than those reported in previous studies of the general female population and female college students [7,26]. Given that the participants were healthcare professionals, this finding suggests that unmarried nurses may be relatively less accepting of EOC as a fertility-preservation strategy. Reproductive decision-making is influenced not only by medical knowledge but also by personal values, social norms, and views about family planning [36]. Social and institutional conditions may also shape attitudes. In South Korea, limited institutional support for EOC and insufficient access to related information may make it difficult for women to perceive EOC as a realistic option [15], and this environment may have affected attitude formation in the present study. Education and counseling should therefore help women understand EOC from a life-course fertility-preservation perspective and make decisions based on adequate information. At the policy level, expanded institutional support and improved access to reliable information are also needed.

Health-related concerns were also significantly associated with EOC intention, with higher levels of concern associated with lower intention. This finding is consistent with previous research showing that women consider potential risks to their own health or the health of future children when making decisions about elective oocyte cryopreservation [7]. In this study, health-related concerns included worries about the health of children born after use of frozen oocytes and concerns that oocyte retrieval or hormonal stimulation might adversely affect women’s own health or future fertility. These concerns may reflect perceived risks related to the safety of ART [38]. Although some studies have reported no significant differences in health outcomes between children born using frozen oocytes and those born using fresh oocytes [38,39], evidence on the long-term health outcomes of children born through EOC remains insufficient. This limited evidence base and residual long-term uncertainty may have contributed to lower EOC intention. Concerns about oocyte retrieval and hormonal stimulation may also act as psychological barriers to ART use [39], consistent with previous findings that trust in the safety of reproductive medical technology is associated with procedure acceptance [16].

Nurses routinely encounter medical procedures, adverse effects, and clinical uncertainty, which may lead them to evaluate procedure-related risks more concretely [40]. A study of female healthcare professionals similarly reported that concerns about the long-term outcomes and safety of oocyte cryopreservation affected intention formation [41]. In this context, the negative association between health-related concerns and EOC intention observed in the present study may reflect risk perceptions shaped by nurses’ clinical experience. However, EOC is no longer considered experimental [9], and studies have reported no significant differences in health outcomes between children born from frozen oocytes and those born from fresh oocytes [39]. Because evidence on long-term health outcomes remains limited, future EOC education and counseling for nurses should present current evidence on oocyte retrieval and fertility, procedural safety, and child health outcomes while also explaining remaining uncertainties. This balanced approach may help nurses make informed reproductive decisions.

Age was also significantly associated with EOC intention, with younger participants reporting greater intention. This finding contrasts with a previous study reporting that the likelihood of considering EOC increases with age [18]. The difference may be attributable to differences in study populations. Because EOC can be understood as a preventive strategy against future fertility decline, younger women may place greater value on its preventive potential and expected benefits. By contrast, older women may approach EOC more cautiously because they must also consider reduced oocyte-cryopreservation effectiveness and the possible need for repeated procedures. Ovarian reserve and oocyte quality decline with age, reducing the probability of pregnancy [42]. Recent studies have also shown that oocyte cryopreservation before 35 years of age may yield a higher likelihood of childbirth with fewer oocytes, whereas older women may need more oocytes to achieve the same delivery outcomes [34]. The number of mature oocytes retrieved also decreases with increasing age at cryopreservation; the median number of mature oocytes retrieved in the first cryopreservation cycle was approximately 15 at 30 years of age, 11 at 35 years of age, and six at 40 years of age [34]. These findings suggest that older age may increase concern about the practical effectiveness and cost-effectiveness of EOC. EOC-related information should therefore be provided at an appropriate time and should explain both the benefits of the procedure and its limitations and potential burdens [43,44]. The higher EOC intention observed among younger participants in this study may reflect the combined influence of these factors. However, because this was a cross-sectional study of unmarried nurses at a single institution, the relationship between age and EOC intention should be reassessed in future studies involving more diverse populations.

Procedural burden, ethical concerns, and EOC knowledge were not significantly associated with EOC intention in this study. This finding differs from previous studies reporting associations between EOC intention and financial burden, ethical considerations, or reproductive knowledge [7,21]. For procedural burden, nurses may have perceived cost as a less important barrier because they had a relatively stable economic foundation [21]. The nonsignificant association with ethical concerns may also be related to the fact that EOC does not directly involve embryo creation [10,13]. EOC knowledge was likewise not significantly associated with intention. Participants’ mean EOC knowledge score was 8.45 points (70.4%), which was higher than scores reported for the general female population (7.90 points, 65.8%) [7] and female college students (6.92 points, 57.7%) [33]. This relatively high knowledge level, together with limited between-participant variability, may explain why EOC knowledge was not clearly associated with intention. In addition, EOC intention may be more closely related to cognitive and psychological factors, such as attitudes toward EOC and perceived health risks, than to knowledge level alone.

This study has several limitations. First, it was a cross-sectional study conducted using convenience sampling at a single institution, which limits the generalizability of the findings and precludes causal interpretation. Second, EOC intention, the outcome variable in the regression analysis, was measured using a single-item VAS. This approach may not have fully captured the multidimensional construct of EOC intention. The associations between EOC intention and its related factors should therefore be interpreted with caution. Third, age was categorized using 30 years as the cutoff because age is clinically important in fertility decline and EOC counseling. In the regression analysis, age was entered as a dummy variable, with participants aged 23–29 years serving as the reference group. Thus, the findings should be interpreted as differences and associations in EOC intention by age category rather than as evidence of a linear association across continuous age. Future studies should clarify the relationship between age and EOC intention using diverse analytical strategies, including models that treat age as a continuous variable. Finally, the internal consistency of the health-related concerns and EOC knowledge scales was somewhat low. Because the health-related concerns scale included only two items, its reliability coefficient may have been affected by the number of items; nevertheless, measurement error may have influenced the observed associations among variables and the interpretation of the findings. Future research should include replication studies and longitudinal studies involving diverse regions and occupational groups to clarify how EOC intention is formed and which factors are associated with it. In addition, reliable and valid multi-item instruments are needed to assess EOC intention multidimensionally, and the reliability and validity of the health-related concerns and EOC knowledge measures should be examined further.

From a practical perspective, the finding that positive attitudes toward EOC were associated with EOC intention indicates the need to provide evidence-based fertility-preservation information early in adulthood. Education and counseling for unmarried nurses should also present current evidence on the medical safety of EOC, the oocyte retrieval process, and long-term health outcomes so that individuals can make informed reproductive decisions.

This study contributes to the literature by identifying EOC intention and its associated factors among unmarried nurses, a group of female healthcare professionals whose reproductive decision-making may be shaped by both occupational and personal considerations. Taken together, the findings indicate that EOC intention is associated with positive attitudes toward EOC, health-related concerns, and age. These results suggest that EOC intention is a multifactorial decision-making process shaped by personal and psychological factors rather than by any single factor alone.

## Figures and Tables

**Table 1.** EOC intention according to participants’ general characteristics (N=159)

Variable	Categories	n (%)	EOC intention		
			Mean±SD	t	*p*
Age (year)	23–29	78 (49.1)	6.11±2.50	3.19	.002
	30–39	81 (50.9)	4.73±2.91		
Education level	Bachelor’s degree	129 (81.1)	5.46±2.81	0.64	.520
	Master’s degree or higher	30 (18.9)	5.10±2.77		
Position	Staff nurse	156 (98.1)	5.38±2.80	–0.38	.710
	Charge nurse, etc.^†^	3 (1.9)	6.00±2.65		
Monthly income (KRW)^‡^	<3 million	89 (56.0)	5.42±2.84	0.13	.900
	≥3 million	70 (44.0)	5.36±2.76		
Religion	Yes	49 (30.8)	5.80±2.49	1.21	.230
	No	110 (69.2)	5.21±2.91		
Experience of OB/GYN visit	Yes	137 (86.2)	5.47±2.89	1.11	.280
	No	22 (13.8)	4.90±2.05		
Reasons for OB/GYN visit (n=137)^§^	Routine check-up	88 (64.2)			
	GYN problem	136 (99.3)			
	OB problem	7 (5.1)			
Results of OB/GYN visit (n=137)^§^	Normal finding	82 (59.9)			
	Endometriosis	9 (6.6)			
	PCOS	31 (22.6)			
	Uterine fibroids	19 (13.9)			
	Others^ǁ^	16 (11.7)			

EOC: Elective oocyte cryopreservation; KRW, Korean won; GYN: gynecology; OB: obstetrics; PCOS: polycystic ovary syndrome.

† Research nurses.

‡ One million KRW was approximately US$713 at the time of data collection.

§ Multiple responses.

ǁ Including ovarian cysts, genital tract inflammation, amenorrhea, etc.

**Table 2.** Descriptive statistics and distributions of EOC-related variables (N=159)

Variable	Possible range	Observed range	Mean±SD	Skewness	Kurtosis
Attitudes toward EOC	11–44	11–44	27.31±6.06	−0.17	0.29
Procedural burden	4–16	4–16	13.47±2.06	−1.08	2.24
Ethical concerns	4–16	4–16	9.49±2.92	0.17	−0.49
Health-related concerns	2–8	2–8	5.73±1.57	−0.42	−0.30
EOC knowledge	0–12	5–12	8.45±1.45	−0.34	−0.22
EOC intention	0–10	0–10	5.38±2.78	−0.57	−0.52

EOC: Elective oocyte cryopreservation.

**Table 3.** Correlations among EOC-related variables (N=159)

Variable	r (*p*)				
	EOC	Procedural burden	Ethical	Health	EOC
	attitude		concerns	concerns	knowledge
EOC attitude	1				
Procedural burden	.04 (.635)	1			
Ethical concerns	.09 (.264)	.12 (.138)	1		
Health concerns	–.11 (.169)	.30 (<.001)	.31 (<.001)	1	
EOC knowledge	–.04 (.595)	.14 (.072)	–.05 (.501)	.32 (<.001)	1
EOC intention	.53 (<.001)	–.09 (.238)	–.02 (.827)	–.26 (.001)	–.01 (.863)

EOC: Elective oocyte cryopreservation.

**Table 4.** Factors associated with EOC intention (N=159)

Variable	B	SE	β	t	*p*
(Constant)	2.39	2.77		0.86	.390
Age, 30–39 years^†^	–1.17	0.44	–.21	–2.64	.009
Education level, master’s degree or higher^†^	0.04	0.53	.01	0.08	.939
Position, charge nurse, etc.^†^	0.00	1.79	.00	0.00	.998
Monthly income, ≥3 million KRW^†^	0.12	0.30	.03	0.40	.692
Religion, yes^†^	–0.15	0.20	–.05	–0.79	.433
Experience of OB/GYN visit, yes^†^	–0.46	0.54	–.06	–0.85	.398
EOC attitude	0.21	0.03	.47	6.82	<.001
Procedural burden	–0.08	0.10	–.05	–0.79	.431
Ethical concerns	0.00	0.07	.00	0.04	.970
Health concerns	–0.42	0.14	–.23	–2.97	.003
EOC knowledge	0.21	0.14	.11	1.51	.132
Durbin-Watson=1.82					
F=7.70 (*p*<*.*001), R^2^=.37, adjusted R^2^=.32					

EOC: Elective oocyte cryopreservation; KRW: Korean won; GYN: gynecology; OB: obstetrics.

† The reference values were as follows: age (23–29 years), education level (bachelor’s degree), position (staff nurse), monthly income (<3 million KRW), religion (no), and experience of OB/GYN visit (no).
